# New applanation tonometer for myopic patients after laser refractive surgery

**DOI:** 10.1038/s41598-020-64013-4

**Published:** 2020-04-27

**Authors:** María Iglesias, Francisco Yebra, Bachar Kudsieh, Andrea Laiseca, Cristina Santos, Jeroni Nadal, Rafael Barraquer, Ricardo P. Casaroli-Marano

**Affiliations:** 1 Instituto Universitario Barraquer, Barraquer Ophthalmology Centre, Barcelona, 08012 Spain; 2 Department of Physics, University of Vigo, 36310 Galicia, Spain; 3 Department of Ophthalmology, Hospital Universitario Puerta De Hierro, 28222 Madrid, Spain; 4 Unitat Antropologia Biològica, Department Biologia Animal, Biologia Vegetal i Ecologia, Universitat Autònoma de Barcelona (UAB), Barcelona, 08193 Spain; 5 International University of Catalunya (UIC), Barcelona, 08017 Spain; 6 Department of Surgery, School of Medicine and Hospital Clinic de Barcelona, University of Barcelona (UB), Barcelona, 08036 Spain

**Keywords:** Biophysics, Medical research

## Abstract

This study assesses the agreement between intraocular pressure (IOP) measurements taken with the Goldmann applanation tonometer (GAT) and a new experimental applanation tonometer with a convexly shaped apex (CT) after laser myopic refractive surgery. Two different CT radii (CT1 and CT2) were designed with a finite element analyser, and a prospective double masked study on 102 eyes from 102 patients was carried out. A Bland-Altman plot and intra-class correlation coefficient (ICC) were calculated to assess the agreement between GAT measurements and the measurements of both CT1 and CT2 before and after myopic laser assisted *in situ* keratomileusis (LASIK; n = 73) and photorefractive keratectomy (PRK; n = 29). We evaluated a subset of two subgroups (n = 36 each) for intra and inter-observer (IA/IE) error. From the whole cohort, the best IOP agreement was observed between GATpre and CT1post surgery: 16.09 ± 2.92 *vs* 16.42 ± 2.87 (*p* < 0.001); ICC = 0.675 (95% CI: 0.554–0.768). In the analysis of LASIK *vs* PRK, GATpre and CT1post showed the highest agreement, although LASIK measurements were more accurate than PRK, as the ICC = 0.718 (95% CI: 0.594–0.812) and ICC = 0.578 (95% CI: 0.182–0.795) respectively. Excellent agreement was observed for IA/IE, and there was an ICC > 0.8 (95% CI) in all cases. CT1 proved more accurate in the LASIK subgroup. In conclusion, our new version of GAT could be used with post-surgery LASIK patients as a more accurate measurement device compared to the current reference tonometer.

## Introduction

The Goldmann applanation tonometer (GAT; Haag-Streit, Switzerland) is the current reference tonometer for measuring intraocular pressure (IOP) in daily clinical practice due to its acceptably accurate measurements, reproducibility and reliability^1–4^. However, GAT readings are influenced by corneal central thickness (CCT) and corneal biomechanics (CB), which vary widely among normal individuals^1,5,6^. In myopic laser refractive surgery (LRS) patients, variations induced in both CCT and CB lead to differences in GAT readings. This change in IOP has been inconsistent in previous studies regardless of the type of surgery: Mardelli *et al*.^7^ found a significant reduction of 1 ± 2.8 mmHg in IOP measurements after ablating 23 μm in photorefractive keratectomy (PRK) patients; while Duch *et al*.^8^ found an underestimation of about 2.9 mmHg per 70 μm ablation in CCT after laser assisted *in situ* keratomileusis (LASIK). These IOP underestimations affect the reliability of GAT as *gold standard* after LASIK and PRK procedures^9–11^.

LASIK is one of the most frequent types of eye surgery and a total of 40 million people had this operation worldwide between 1991 and 2016^12^. Moreover, the prevalence of glaucoma is 2 to 4 times higher among the myopic population^13^. Thus, it is very important to obtain an accurate and real estimation of IOP in order not to miss ocular hypertension after myopic LRS.

The aim of our study is to describe a new modified GAT as well as its translational application and clinical outcomes by evaluating IOP measurements before and after myopic LRS. We also evaluate the reliability of the intra and inter-observer agreement.

## Methods

### New device description

**Figure 1:**
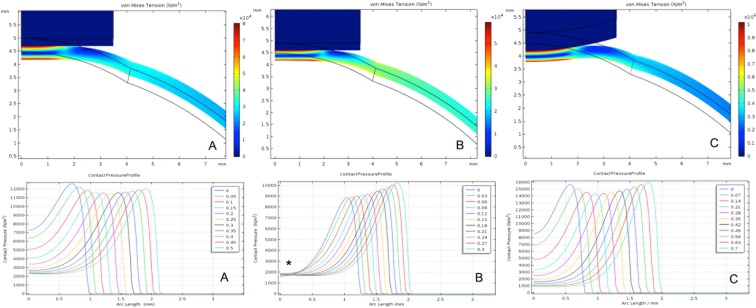
FEA simulation of corneal antero-posterior displacement in response to GAT (**A,B**) and CT (**C**) contact. The graphics below correspond to force applied from the centre to the periphery of the cornea within the anterior tonometer contact surface, and the colour scale indicates the maximum corneal deformation (MCD = N/m^2^). A corresponds to CC. B and C correspond to OC with a 100 μm ablation of CCT. A similar arc length and a wider contact pressure profile graphic are observed when GAT contact is compared with normal corneas (**A**), and CT contact is compared with operated corneas (**C**). However, when GAT is used on an OC (**B**), a confluence of forces is observed at the same point (asterisk, *) from the beginning, and the arc length contact is narrower. Young’s Modulus (Y) = 0.5 MPa. CC, calibrated cornea. OC, operated cornea.

How corneal tissue will react in real life to external elements can be estimated with finite element analysis (FEA). Three-dimensional (3D) modelling is more accurate than two-dimensional (2D) modelling for predicting what could occur to tissue *in vivo*^14^. In our study, we used 2D (Fig. 1) and 3D (Fig. S1, supplementary data) FEA to simulate the biomechanical responses of a normal cornea and a laser operated cornea (OC) to the contact of two external forces: a plane surface (corresponding to GAT) and a convex surface (corresponding to the new CT device). Various simulations were carried out with different corneal thicknesses and elastic behaviours of the cornea (Figs. S2–S4).

**Figure 2:**
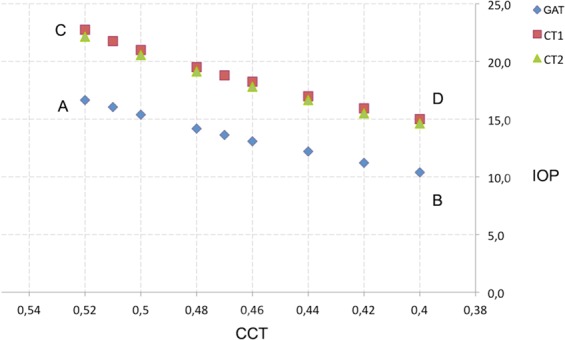
The FEA simulation showing the IOP behaviour response to the ablation of CCT measured with the 3 different tonometer devices (Young’s modulus = 0.5 MPa). CCT, corneal central thickness; IOP, intraocular pressure; GAT, Goldmann applanation tonometer; CT1-CT2, convex tonometers.

Two different individual corneas were designed. First, a regular or “calibration cornea” (CC), as described in the Orssengo-Pye algorithm^6^, with CCT = 520 μm, an anterior corneal curvature of 7.8 mm, and assuming radial symmetry for a cornea-GAT contact of 3.06 mm (Fig. 1A). The second FEA consisted in a “LRS OC”, whose CCT and anterior radial symmetry could be ablated as in LRS up to 420 µm and 8.43 mm for different simulations (Figs. 1B,C and 2).

In all cases, a nonlinearity material model with Mooney-Rivlin parameters^15^ was applied, and every simulation was calculated with Young’s Modulus (Y) = 0.5 MPa. Inverse modelling was performed to account for the IOP: a step-wise computing initial stress state of the cornea was determined by assuming IOP = 15 mmHg^16^.

Due to pachymetric and elastic corneal variability in our population, we considered different radii for the CT devices (radii varying from 11.5 to 14 mm) to compare contact pressure profiles (CPP) with different corneas, until a similar corneal modelling behaviour was observed between GAT and CT (Fig. 1A,C).

**Figure 3:**
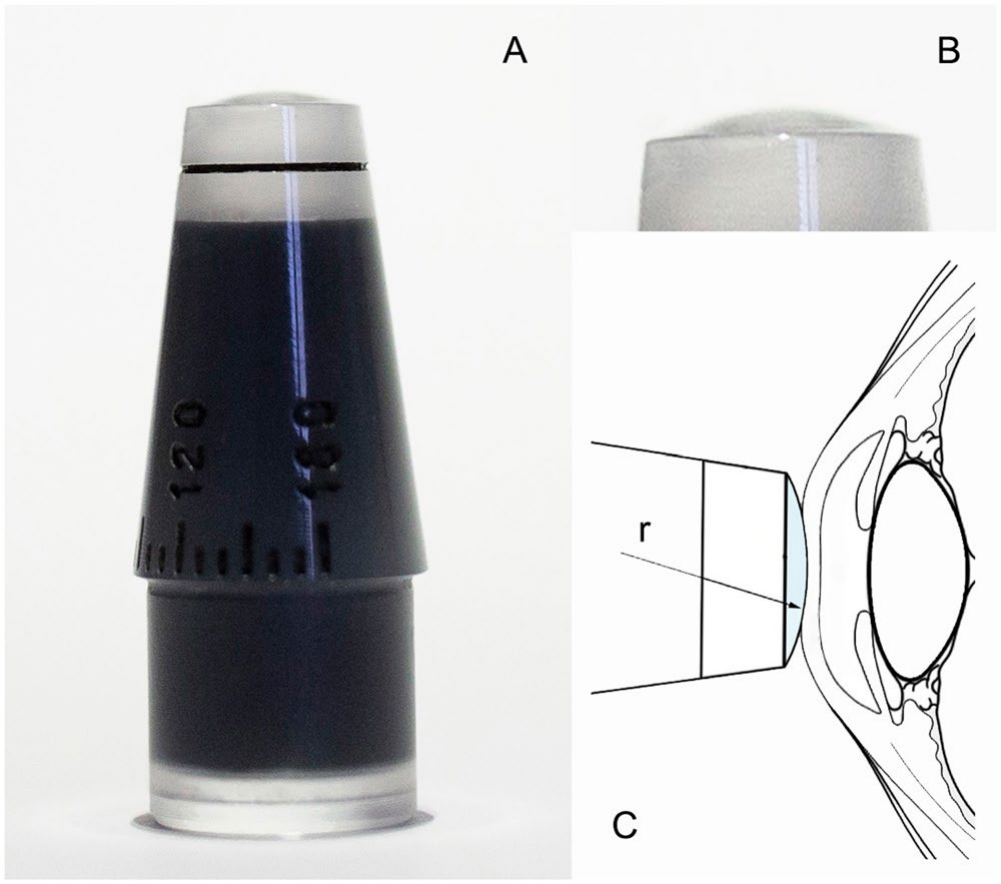
New modified CT applanation tonometer (**A**). Detail of the convexed apex (**B**). Diagram representing cross section of CT showing its radius (r) in contact with an LRS operated cornea (**C**).

Finally, two different tonometers with different radii (r) (Fig. 3) were created and called CT1 (r = 13 mm) and CT2 (r = 12 mm). These were used in our clinical study to assess which tonometer correlated better with GAT measurements before LRS.

#### Study design

A prospective double-masked comparative study was carried out on a sample of 102 myopic subjects who were going in for LSR (LASIK or PRK) at the Barraquer Ophthalmology Center in Barcelona. These subjects first underwent a general medical history review, and a detailed eye examination. After meeting the inclusion criteria and having none of the exclusion criteria, the subjects were included and their informed consent was obtained for study participation. The study protocol is in accordance with the Declaration of Helsinki and the Institutional Ethics Committee of the Barraquer Ophthalmology Center approved this study.

#### Subjects

The right eye of each subject was randomly selected adding up to a total of 102 eyes. The inclusion criteria were: myopic patients aged above 18 years with a stable myopic refractive error less than spherical −9 diopters (dpts), and less than myopic astigmatism −4 dpts. Subjects who had previously been diagnosed with an ocular pathology before ocular surgery or treated with medications that could affect IOP levels were excluded from the study.

#### Exploration protocol

All patients underwent a standardized examination that included measuring visual acuity (spherical equivalent refraction; SER), slit-lamp anterior biomicroscopy, posterior segment ophthalmoscopy and IOP measurements. Age, gender and refractive error were also recorded.

Before IOP measurements were recorded, the corneal topography was determined with a Pentacam (Oculus, Wetzlar, Germany) to obtain anterior simulated keratometry and posterior corneal curvature (simK, PCC respectively), corneal volume (VOL) and CCT. In addition, the corneal hysteresis (CH) and corneal resistance factor (CRF) were obtained by Ocular Response Analyser (ORA; Reichert Ophthalmic instruments, New York). The maximum ablation depth (Max.Abl) and percentage of ablated tissue (PTA) were also analysed. Postoperative treatment included ofloxacin 0.3% and fluorometholone (FML) 0.1% 4 times a day for 1 week in both groups, and an additional 3 weeks of FML in the PRK group.

IOP was measured before and three months after surgery. Each measurement was carried out by the main observer (MI) using 3 different devices (GAT, CT1, CT2 pre- and post- surgery, used in the same order, respectively) and leaving 2 minutes between taking measurements with each tonometer^17^. The IOP measurements were taken between 10:00 and 13:00 hours (10 am and 1 pm), similarly to the 3^rd^ month after surgery readings, which were performed at approximately the same time as the pre-operation readings^18,19^.

In order to assess intra-observer (IA) error, a subset of 36 patients were randomly double-masked measured by the main observer MI in the presence of a second observer (AL). To assess the inter-observer (IE) error, a different subset of 36 patients were also evaluated randomly by the main observer MI and by a second observer AL. In these cases, IOP measurements were carried out consecutively, were double-masked and after an interval of 5 minutes. In all cases, a third observer (BK) was present to ensure the double-masking.

#### Statistical analysis

The statistical analysis was performed using SPSS^®^ (Statistical Package for Social Sciences, v22.0; SPSS Inc., Chicago, IL, USA). A significance level of 5% was considered in all the analyses. All quantitative variables were tested for normality with the Kolmogorov-Smirnov or the Shapiro-Wilk tests. We conducted descriptive analyses for all the variables preoperatively and 3 months after surgery. The descriptive values are presented as mean ± standard deviation unless stated otherwise.

Pre- and post-surgery values were compared considering all the cases using the paired *t*-test or the Wilcoxon test depending on data normality. In addition, we compared the eye characteristics of patients undergoing surgery with LASIK. PRK was performed using the independent *t*-test or the Mann-Whitney test depending on data normality.

To evaluate the relationship between IOP and the corneal characteristics, IOP values obtained with GAT, CT1 and CT2 in pre- and post-surgery were correlated respectively with clinical characteristics of pre- and post-surgery eyes using the Pearson or Spearman’s correlation coefficients depending on data normality.

We considered GATpre as the current reference for evaluating the concordance between IOP measures taken with the different tonometers. Different approaches were used to evaluate the differences between GATpre IOP and IOP determined with GAT, CT1 and CT2post respectively. In short, 1) the mean differences between GATpre measurements and the measurements taken with all the devices in post-surgery were calculated and the absence of differences was tested with the Wilcoxon test; 2) the Bland-Altman analysis was used to compare the agreement of measurements taken with the different tonometers; and 3) the ICC was calculated based on absolute-agreement. Values lower than 0.5, between 0.5 and 0.8, and greater than 0.8 were indicative of poor or weak, good, and excellent reliability, respectively^20^. To determine which factors explain the bias trend in the Bland-Altman analysis, multiple stepwise regression was used to relate the differences in IOP and the change in corneal characteristics from pre- to post-surgery.

To analyse IA/IE, we calculated the mean difference between measurements and tested the absence of differences with the Wilcoxon or paired sample *t*-test. The Bland-Altman analysis was used, and the ICC was calculated based on absolute-agreement.

## Results

### FEA simulation results

CPP comparisons can be helpful for understanding how corneal tissue will react to contact with different tonometers, such as GAT or CT. To compare these behaviours easily, we developed a 2D graphic diagram in which a cross sagittal section of corneas and tonometers are shown with their corresponding graph below (Fig. 1).

When the flat area of GAT contacts a CC (Fig. 1A), an initial arc length rising from the centre is observed with an initial contact pressure (ICP; blue line) = 7350 N/m^2^. The maximum corneal deformation is (MCD) = 8 × 10^4^ N/m^2^. This contrasts with a lower initial arc length converging in the centre that is observed if the GAT contacts an OC, and its ICP = 1600 N/m^2^ (Fig. 1B; blue line, asterisk) and MCD = 6 × 10^4^ N/m^2^. However, the OC response recovers similarly to its original (from 1600 to 8500 N/m2) when an external force with a convex surface (CT) contacts the anterior corneal surface after surgery (Fig. 1C), and its MCD = 1 × 10^5^ N/m^2^. On the other hand, when CT is applied to a CC (Fig. S4,A), these values increase compared to GAT values in normal cornea ICPs of 26000 N/m^2^.

The CPP barely changed when different elastic behaviours were introduced in a normal cornea (Fig. S2A,B). However, the CPP changed considerably with different thicknesses and when Y varied after the CCT was ablated (Figs. S2C,D–S4).

Another important element of FEA is to evaluate how pachymetry variations influence IOP estimations. With a CC (CCT = 520 μm) with an initial IOP of 15 mmHg (Fig. 2A), as the CCT was ablated we observed a linear reduction in the GAT IOP measurements (Fig. 2B) (−4.4 mmHg for a corneal ablation of 80 μm). However, an overestimation of IOP was recorded when a CC was measured with CT1pre (+6.1 mmHg) and CT2pre (+5.5 mmHg) (Fig. 2C). The same IOP underestimation was recorded parallel to GAT values when CCT was ablated and IOP was measured with CT1 or CT2: −5.7 and −5.5 mmHg, for a corneal ablation of 80 μm, respectively (Fig. 2D).

### Clinical results

#### Demographic information and pre- and post-surgical eye characteristics

A total of 102 eyes from 102 patients (61.8% male; n = 63) were included in the study. Seventy-three (71.6%) patients underwent LASIK and 29 (28.4%) PRK. The mean age was 31.6 ± 6.1 years. The descriptive statistics of variables in the pre- and post-surgical evaluation are normally distributed and are shown in Table 1.

**Table 1:** Descriptive statistics of variables in the pre- and post-surgical evaluation 3 months later.

	PRE-SURGERY MD ± SD	POST-SURGERY MD ± SD
SER		
• ALL*	−4.23 ± 2.15	0.01 ± 0.24
• LASIK	***−4.59*** ± ***2.31***	−0.02 ± 0.26
• PRK	***−3.31*** ± ***1.34***	0.00 ± 0.21
simK		
• ALL^#^	43.72 ± 1.22	40.01 ± 2.16
• LASIK	43.60 ± 1.26	***39.70*** ± ***2.27***
• PRK	44.00 ± 1.08	***40.77*** ± ***1.67***
VOL		
• ALL^#^	61.49 ± 3.52	59.77 ± 3.65
• LASIK	***62.28*** ± ***3.51***	***60.56*** ± ***3.58***
• PRK	***59.50*** ± ***2.68***	***57.77*** ± ***3.02***
PCC		
• ALL^#^	6.34 ± 0.20	6.37 ± 0.20
• LASIK	6.34 ± 0.19	6.37 ± 0.19
• PRK	6.34 ± 0.23	6.37 ± 0.21
CCT		
• ALL^#^	549.91 ± 32.04	471.79 ± 42.64
• LASIK	***559*** ± ***29.94***	476 ± 44.10
• PRK	***526*** ± ***25.02***	459 ± 36.29
CH		
• ALL^#^	10.93 ± 1.49	8.42 ± 1.43
• LASIK	11.06 ± 1.51	8.54 ± 1.45
• PRK	10.62 ± 1.64	8.11 ± 1.36
CRF		
• ALL*	10.37 ± 1.68	6.90 ± 1.58
• LASIK	10.56 ± 1.57	6.86 ± 1.65
• PRK	9.89 ± 1.40	7.00 ± 1.39
PTA		
ALL		12.02 ± 4.59
LASIK		***12.73*** ± ***4.77***
PRK		***10.23*** ± ***3.57***
Max.Abl		
• ALL		66.28 ± 26.18
• LASIK		***71.23*** ± ***27.19***
• PRK		***53.83*** ± ***18.60***
GAT*		
• ALL	16.09 ± 2.92	12.52 ± 2.44
• LASIK	15.99 ± 2.95	***12.04*** ± ***2.17***
• PRK	16.38 ± 2.89	***13.76*** ± ***2.16***
CT1*		
• ALL	22.45 ± 4.00	16.42 ± 2.87
• LASIK	22.36 ± 4.15	***15.80*** ± ***2.60***
• PRK	22.69 ± 3.66	***18.00*** ± ***2.65***
CT2*		
• ALL	23.49 ± 4.02	17.01 ± 2.87
• LASIK	23.53 ± 4.32	***16.41*** ± ***2.45***
• PRK	23.38 ± 3.22	***18.52*** ± ***3.30***

SER, spherical equivalent refraction; simK, anterior simulated keratometry; PCC, posterior corneal curvature, CCT central corneal thickness, PTA percent tissue altered, Max.Abl maximum corneal ablation, CH corneal hysteresis, CRF corneal resistance factor, VOL corneal volume, IOP intraocular pressure. GAT Goldmann applanation tonometer, CT1-CT2 convex tonometer.

Comparison between pre- and post-surgery considering all the cases: ^*^Wilcoxon test, *p* < 0.001; ^#^Paired *t*-test, *p* < 0.001.

All values are in mean ± standard deviation (MD ± SD). Significant differences between LASIK and PRK are marked in cursive bold.

#### Relationship between IOP and corneal characteristics

IOP values obtained with GAT, CT1 and CT2 in pre- and post-surgery show a significant correlation with CRF for all patients (Table 2). The post-surgery IOP determined with any applanation device correlates with post-surgery CRF, simK, Max.Abl and PTA (Table 2). In addition, CT1 IOP measures correlate with CH.

**Table 2:** Relationship between IOP and corneal characteristics for all eyes (n = 102) in pre- and post-surgery.

		PRE			POST		
		GAT	CT1	CT2	GAT	CT1	CT2
CH	R	0.067	0.130	0.118	0.157	***0.241***	0.194
	p	0.502	0.193	0.237	0.116	*0.015*	0.051
CRF	R	***0.380***	***0.453***	***0.427***	***0.428***	***0.491***	***0.443***
	p	*<0.001*	*<0.001*	*<0.001*	*<0.001*	*<0.001*	*<0.001*
simK	R	0.176	0.135	0.128	**0.256**	**0.230**	*0.266*
	p	0.077	0.177	0.200	0.009	0.020	0.007
CCT	R	0.115	***0.201***	***0.268***	0.102	0.112	0.139
	p	0.249	*0.043*	*0.006*	0.305	0.263	0.164
SER	R	−0.110	−0.157	***−0.211***	0.012	−0.073	−0.080
	p	0.270	0.116	*0.033*	0.909	0.467	0.425
VOL	R	0.064	0.149	***0.261***	−0.057	0.033	0.028
	p	0.523	0.135	*0.008*	0.568	0.741	0.776
PCC	R	−0.142	−0.111	−0.146	−0.081	−0.113	−0.142
	p	0.154	0.268	0.144	0.419	0.260	0.155
PTA	R				***−0.237***	***−0.244***	***−0.292***
	p				*0.016*	*0.013*	*0.003*
Max.Abl	R				***−0.250***	***−0.241***	***−0.287***
	p				*0.011*	*0.015*	*0.003*

SER, spherical equivalent refraction; simK, simulated keratometry; PCC, posterior corneal curvature, CCT central corneal thickness, PTA percent tissue altered, Max.Abl maximum corneal ablation, CH corneal hysteresis, CRF corneal resistance factor, VOL corneal volume, IOP intraocular pressure. GAT Goldmann applanation tonometer, CT1-CT2 convex tonometer. R- Correlation coefficient; *p* -value for correlation.

Comparison between pre- and post-surgery considering all the cases: Pearson or Spearman’s correlation coefficient, depending on the data normality. Present significant differences are marked in cursive bold.

Considering LASIK and PRK patients separately, in the LASIK subgroup CCT, CRF and PTA are correlated with values of IOP in post-surgery for all tonometers. However, in PRK patients, IOP is only related to pre- and post-surgery CRF (table not shown).

#### IOP evaluation using different tonometer devices

**Figure 4:**
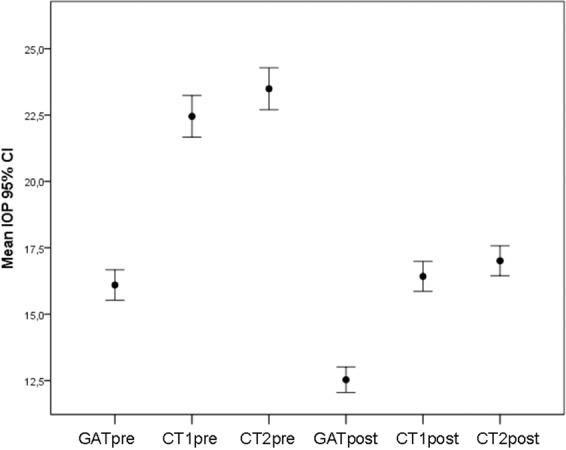
Mean IOP (95% CI: confidence interval) obtained using different tonometer devices in pre- and post-surgical evaluations. GAT, Goldmann applanation tonometer; CT1-CT2, convex tonometers.

We evaluated IOP for different tonometers by analysing the concordance of the CT1 and CT2post measurements. Considering GATpre the main reference, CT1 and CT2 in pre-surgery appear to overestimate IOP (Fig. 4). However, in post-surgery, GAT underestimates the IOP, and CT1 and CT2 provide similar values to those obtained with GATpre. A similar result is obtained if LASIK and PRK patients are considered independently (Fig. S5). For all patients, the IOP values measured with different devices in post-surgery evaluation are different, and the IOP obtained with GATpre has a value near to zero similarly to CT1post (mean difference 0.32 mmHg), whereas the mean value is significantly different from zero for GATpost (−3.56 mmHg) and CT2post (0.91 mmHg) (Table 3).

**Table 3:** Mean difference (bias) for IOP measurements in pre-surgery with GAT and in post-surgery with GAT, CT1 and CT2.

	IOP difference	GATpost - GATpre	CT1 - GATpre	CT2 - GATpre
All	Mean difference (bias)	***−3.569***	***0.324***	***0.912***
	Wilcoxon test	Z = −8.618; *p* < *0.001*	Z = −1.318; *p* = 0.187	Z = −3.523; *p* < *0.001*
	Regression bias	b = −0.464; *p* < *0.001*	b = −0.336; *p* < *0.001*	b = −0.362; *p* < *0.001*
LASIK	Mean difference (bias)	***−3.9452***	***−0.192***	***0.425***
	Wilcoxon test	Z = −7.383; *p* < *0.001*	Z = −0.823; *p* = 0.410	Z = −1.687; *p *= 0.092
	Regression bias	b = −0.528; *p* < *0.001*	b = −0.364; *p* < *0.001*	b = −0.416; *p* < *0.001*
PRK	Mean difference (bias)	***−2.621***	***1.621***	**2.138**
	Wilcoxon test	Z = −4.373; *p* < *0.001*	Z = −3.16; *p* = *0.002*	Z = −3.469; *p* = *0.001*
	Regression bias	b = −0.346; *p* = *0.010*	b = −0.327; *p* = *0.035*	b = −0.283; *p* = 0.108

GAT, Goldmann applanation tonometer; CT1-CT2, convex tonometer; IOP intraocular pressure. Z, Wilcoxon test;b, regression coefficient; p, probability value.

Mean difference between measures with GATpre and all the devices in post-surgery were computed and tested for the absence of differences with the Wilcoxon test.

The results of regression bias (related to Bland-Altman) are also shown. Present significant differences are marked in cursive bold.

**Figure 5:**
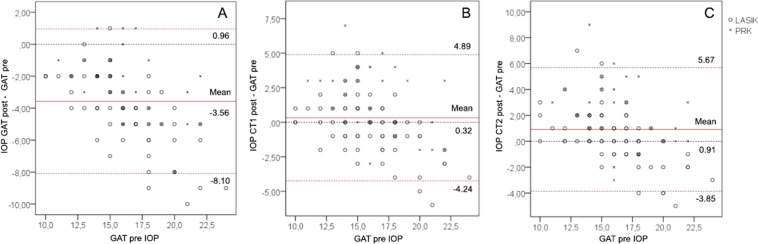
Bland-Altman analysis (n = 102) comparing the relation between GATpre and GATpost (**A**), CT1post (**B**) and CT2post (**C**). Continuous red line: observed mean difference; dotted red lines: lower and higher limits of agreement; dotted black line: mean difference of zero. GAT, Goldmann applanation tonometer; CT1-CT2, convex tonometers.

Considering LASIK and PRK patients separately, CT1post (−0.19 mmHg) showed the smallest differences with GATpre (1.62 mmHg) for both LASIK and PRK (Table 3). Considering LASIK and PRK patients together, the Bland-Altman analysis showed poor agreement between GATpre and GATpost (Fig. 5A) (mean differences: −3.56 mmHg, *p* < 0.001; limits of agreement: −8.10–0.96). However, a much better agreement is observed between GATpre and CT1post (Fig. 5B) (mean differences: 0.32 mmHg, *p* = 0.187; limits of agreement: −4.24–4.89) and CT2post (Fig. 5C) (mean differences: 0.91 mmHg, *p* < 0.001; limits of agreement: −3.85–5.67).

In the LASIK group, GATpost proved to have a poor agreement (mean differences: −3.94 mmHg, *p* < 0.001; limits of agreement: −8.42–0.50). A better agreement was found for CT1 (mean differences: −0.19 mmHg, *p *= 0.410; limits of agreement: −4.30–3.92) and CT2post (mean differences: −0.42 mmHg, *p* = 0.092; limits of agreement: −3.77–4.62) respectively. The PRK group also showed poor agreement for GATpost (mean differences: −2.62 mmHg, *p* < 0.001; limits of agreement: −6.79–1.55). A better agreement was found for CT1post (mean differences: 1.62 mmHg, *p* = 0.002; limits of agreement: −0.70–2.54) and CT2post (mean differences: 2.13 mmHg, *p* = 0.001; limits of agreement: 1.11–2.16) (Table 3).

Nevertheless, in all cases a bias trend was observed. Bland-Altman analysis and the regression bias results for LASIK and PRK patients show best agreements for CT1 followed by CT2. To determine which factors explain the bias trend, multiple stepwise regression was used to relate the IOP differences and the change in corneal characteristics from pre- to post-surgery. For all patients and for subgroups (LASIK and PRK), differences between GATpre and the measurements in post GAT, CT1 and CT2 appeared to be correlated with the differences in CRF before and after surgery (*p* < 0.001) (Table 2).

The ICC, calculated for all the patients and considering LASIK and PRK separately, had poor or moderate agreement between pre- and post-surgery GAT measurements. However, a good agreement was observed for CT1 and CT2, and the highest values were obtained when the agreement was tested between GATpre – CT1post (*p* < 0.001) (ICC = 0.718; 95% CI: 0.594–0.812) in the LASIK soubgroup (Table 4).

**Table 4:** Intraclass correlation coefficient (ICC) results.

	Tonometer pair	n	CI (95% CI)	*p*
All patients	GATpost- GATpre	102	***0.338*** (−0.100–0.656)	*<0.001*
	CT1post - GATpre	102	***0.675*** (0.554–0.768)	*<0.001*
	CT2post - GATpre	102	***0.621*** (0.459–0.738)	*<0.001*
LASIK	GATpost - GATpre	73	***0.284*** (−0.096–0.614)	*<0.001*
	CT1post - GATpre	73	***0.718*** (0.594–0.812)	*<0.001*
	CT2 post - GATpre	73	***0.684*** (0.540–0.789)	*<0.001*
PRK	GATpost - GATpre	29	***0.494*** (−0.081–0.789)	*<0.001*
	CT1post - GATpre	29	***0.578*** (0.182–0.795)	*<0.001*
	CT2 post- GATpre	29	***0.509*** (0.065–0.762)	*<0.001*

GAT, Goldmann applanation tonometer; CT1-CT2 convex tonometer.

CI, confidence Interval; *p*, value for comparison.

ICC was based on absolute-agreement. Present significant differences are marked in cursive bold.

#### Intra and inter-observer errors

In terms of the IE of the measurements between the main and second observer in 36 patients pre- and post-surgery, mean differences between measurements performed with several devices do not show significant differences (Table 5). In all cases, the ICC is higher than 0.8 (*p* < 0.001), and the values are lower in the postoperative period: GATpost ICC = 0.833 (95% CI: 0.697–0.911), CT1post ICC = 0.857 (95% CI: 0.738–0.924), and CT2 post ICC = 0.833 (95% CI: 0.698–0.911). Concerning IA error measurements, in all cases the ICC is higher than 0.9 (*p* < 0.001) and the values are lower in the postoperative period: GATpost ICC = 0.948 (95% CI: 0.895–0.975), CT1post ICC = 0.933 (95% CI: 0.866–0.967), and CT2post ICC = 0.966 (95% CI: 0.927–0.984) (Table 6).

**Table 5:** Inter-observer error between the main and second observer (n = 36).

	Mean (bias)	Mean 95%CI	Standard deviation- sd	Lower LE = Mean-1.96*sd	Higher LE = Mean+ 1.96*sd	Regression bias
M GAT pre - L GAT pre	0.028	−0.364–0.420	1.158	−2.243	2.298	b = −0.014; *p *= 0.829
M GAT post - L GAT post	0.056	−0.372–0.483	1.264	−2.421	2.532	b = −0.154; *p *= 0.137
M CT1 pre - L CT1 pre	0.472	−0.152–1.096	1.844	−3.141	4.086	b = 0.000; *p* = 0.999
M CT1 post - L CT1 post	0.333	−0.165–0.832	1.474	−2.555	3.222	b = −0.089; *p* = 0.346
M CT2 pre - L CT2 pre	0.028	−0.493–0.549	1.540	−2.990	3.046	b = −0.045; *p* = 0.493
M CT2 post - L CT2 post	−0.389	−0.968–0.190	1.712	−3.744	2.966	b = −0.183; *p* = 0.069

GAT, Goldmann applanation tonometer; CT1-CT2, convex tonometer; M, main observer; L second observer. LE, limit of agreement; *p*, probability value for regression; b, regression coefficient.

The mean difference between measurements was computed and the absence of differences was tested with the Wilcoxon or paired sample *t*-test; the Bland-Altman analysis was used; and the ICC was calculated based on absolute-agreement.

Present significant differences are marked in cursive bold.

**Table 6:** Intra-observer error between the first and second measurement (n = 36).

	Mean (bias)	Mean 95%CI	Standard deviation- sd	Lower LE = Mean-1.96*sd	Higher LE = Mean+ 1.96*sd	Regression bias
GAT1st pre-GAT2nd pre	0.000	−0.304–0.304	0.649	−1.272	1.272	b = 0.010; *p *= 0.836
GAT1st post - GAT2nd post	−0.161	−0.375–0.053	0.583	−1.304	0.981	b = −0.051; *p* = 0.392
CT11st pre - CT12nd pre	−0.368	−0.930–0.193	1.165	−2.651	1.915	b = −0.006; *p* = 0.912
CT1 1st post - CT1 2nd post	0.000	−0.328–0.328	0.894	−1.753	1.753	b = −0.095;* p* = 0.171
CT2 1st pre - CT2 2nd pre	0.150	−0.125–0.425	0.587	−1.001	1.301	b = 0.011; *p *= 0.721
CT2 1st post - CT2 2nd post^#^	***−0.258***	−0.508–0.008	0.682	−1.594	1.078	b = 0.052; *p* = 0.257

GAT, Goldmann applanation tonometer; CT1-CT2, convex tonometer.

LE- Limit of agreement. ^#^*p *< 0.05.; *p*, probability value for regression; b, regression coefficient.

The mean difference between measurements was computed and the absence of differences was tested with the Wilcoxon or paired sample *t*-test; the Bland-Altman analysis was used; and the ICC was calculated based on absolute-agreement. Present significant differences are marked in cursive bold.

## Discussion

The applicability of the Imbert-Fick law for applanation tonometry is compromised after myopic LRS^3,5,8,21–23^. CB modifications after LRS alter CPP with GAT, so that the corneal force exerted from the centre is much lower in LRS operated eyes compared to standard corneas. We have confirmed that the flattened centre of an OC (3.06 mm area of applanation) is consistent with the idea of Imbert-Fick behaviour^24^, but not the edges when the GAT is applied. Shih^23^ also described the area of highest stress to be located around the ablated edge under applanation when CB and IOP were compared between LASIK and PRK. However, we have demonstrated that a different phenomenon can be observed when a convex force (CT) is applied towards the centre of an ablated zone: the initial contact pressure increases in the centre, resulting in a balance of forces similar to that which existed before surgery.

Multiple theoretical models have been described with very different Y estimations (0.1–1.24 MPa)^5,6,23,25^. Hamilton^6^ found a 5.35 mmHg error caused by an increase from 0.16 MPa to 0.40 MPa, and a 4.67 mmHg IOP difference across the CCT sample (487.7 to 599.9 μm). This means that both Y and CCT influence IOP measurements *in vivo* estimations. In our Y = 0.5 MPa *ex vivo* calculations we found some mathematical results that, without being absolute, correspond to what was obtained in the clinical study: FEA analysis showed that GATpost recorded a −4.4 mmHg as CCT was ablated 80 μm. Considering that LASIK reduces CCT more than PRK (with Max.Abl = 71.23 μm in our sample) this value nearly corresponds to the GATpost underestimation of −3.94 mmHg shown in our LASIK results.

The FEA analysis of CT1post and CT2post compared to GATpre showed IOP differences of 0.4 and 0.0 mmHg respectively, which correlates similarly to our clinical results of a CT1post of −0.19 mmHg and a CT2post of 0.42 mmHg. According to these comparisons, this should have a large impact on the model for explaining what may be truly measured in real life. Hence, these simulations seem valid to compare differential behaviours among different tonometers or between corneas with different thicknesses.

However, we must make a conservative interpretation. FEA has several limitations, mainly due to the complexity of representing ocular tissues mathematically, which affects the range of applicability of the Imbert-Fick law. Furthermore, from a mechanical point of view, the structural geometry of the eye cannot be perfectly represented as it is limited by the numerical viability of the simulation. FEA simulations are complex and require multiple models to achieve hypothetical geometric pre- and postoperative corneas^26^. The main limitation of our numerical models is that they do not take into account the viscoelastic behaviour of the cornea. In addition, they were designed for PRK and not LASIK. Nevertheless, previous results^23^ have found similar corneal deformation patterns in 3D and 2D FEA between PRK and LASIK. Therefore, the two surgical approaches are comparable in terms of central corneal displacement. Since our goal was not to compare different corneal biomechanics between LASIK and PRK, but rather to evaluate areas of applanation (corneal displacement) contacted by different external tonometers after LRS, we decided to simplify our comparisons and use a PRK shaped cornea.

Besides, another significant limitation still affects the tonometry: the internal variability in corneal idiosyncrasies. As discussed previously^27^, it is almost impossible to find a single universal number for corneal clinical properties. Assessing how the strength or weakness of a cornea can influence IOP readings seems unrealistic beyond a single pachymetric or biomechanical parameter. This implies that tonometry is not reliable, not only due to CCT variability^28^, but also due to the alteration of the corneal structure. Therefore, correction algorithms for GAT are not reliable since they are based only on changes in CCT^6,29^.

It has been described^30–32^ that the bias in internal patient variability in CCT can be minimized with modified versions of GAT, which opens a new horizon for GAT modifications in the near future. Mccafferty *et al*. demonstrated that a bi-curved concave-convex surface modified GAT can significantly reduce GAT prism sensitivity to CCT in standard corneas. However, our FEA analysis and clinical results show that a central convex modification of GAT seems to be accurate for measuring IOP in OCs. Therefore, this study is the first to show that a unique convex surface modified version of GAT could be reproducible for post operated LASIK or PRK IOP measurements.

Concerning other devices, the Pascal tonometry (PDCT) does not appear to be as influenced by CB after LASIK as other tonometers^33^. After reducing the mean corneal pachymetry of 90.2 μm, Pepose *et al*. found no statistically significant differences in pre- and post-surgery IOP measurements taken with PDCT (−0.5 ± 2.6 mmHg, *p* = 0.27**)**, compared to pre-and post-measurements taken with GAT (−1.8 ± 2.8 mmHg *p* < 0.01). Sales-Sanz^34^ found that the Schiøtz tonometer has less disparity in terms of coefficient ocular rigidity (*Ko*) than PDCT and GAT after the LASIK procedure, although it is not commonly used in clinical practice. There are other devices that are more extensively used, such as the rebound tonometer iCare (Tiolat Oy, Helsinki, Finland); however, it underestimates IOP in post LASIK like GAT. Previous reports^35^ described no statistically significantly difference between iCare and GAT in 96 myopic patients after LASIK with a mean underestimation of −4.9 ± 2.5 mmHg and −3.4 ± 2.5 mmHg, respectively.

With respect to tonometers that are less influenced by CB^36^, different IOP measurements between the Corvis ST tonometer, GAT and ORA (corneal compensated IOP and Goldmann-correlated IOP) have been described in 50 myopic patients after LASIK: 3.4, 1.0 and 3.8 mmHg Bland-Atlmann bias respectively (95% limits of agreement of −0.7 to 7.5, −2.1 to 4.2, and −0.4 to 8.0 mmHg). In our LASIK subgroup of 73 patients, the mean difference for CT1post was 0.19 mmHg (limits of agreement: −4.30–3.92), and 0.42 mmHg for CT2post (limits of agreement: −3.77–4.62), indicating that IOP estimations with CT1post are similar to those provided with GATpre. Therefore, it seems that CT1 offers a more accurate estimation in patients with LASIK than GAT. Nevertheless, tonometry after LRS may not be interchangeable due to the diversity in the results. We believe it is relevant which tonometer and which IOP are taken as baseline after LRS, and that GATpost should not be taken as IOP reference. Our new device correlated with GATpre in our LASIK group, but an important remark of our study is that GATpre is an estimation of IOP and could not be accurate. Besides, CT must not be used in subjects whose CB properties have not been strongly modified by laser -as in PRK corneas- because it seems to overestimate IOP. On the other side, we have not compared it with other non-applanation tonometers in pre or post-surgery. Future studies are necessary to evaluate agreement between CT and PDCT, Corvis ST, or corneal compensated IOP in this subgroup of patients.

In contrast to the LASIK group, our PRK patients showed greater deviation, indicating that the CT device is not as accurate as for LASIK patients. In our Bland-Altman analysis, the IOP readings obtained with CT1post were similar to those obtained with GATpre for all patients. However, for PRK patients the deviation was higher than in LASIK patients, indicating that CT performed less accurately for the PRK approach. Moreover, GATpost showed a relatively good agreement in the PRK group, which means GAT could be reproducible in PRK patients. This is consistent with previous research^7^, which found minimal changes with GAT (0.5 ± 2.4 mmHg; *p* < 0.01) in 111 PRK patients with a mean ablation of 23 ± 23 μm in corneal thickness. Furthermore, the PRK group received FML during 4 weeks after surgery. Despite topical steroids (TS) could have elevated IOP^37^, we believe that differences in IOP in both groups are due to CB changes, not to TS effect. FML has proven to be less ocular hypertensive than other topical steroids^37^, and TS effect on IOP recovers to baseline within 1 to 3 weeks of discontinuing treatment^38^. Our PRK IOP was measured after 8 weeks of stopping TS, so it seems steroids could not influence the IOP measurements at this point.

Other limitations affect our research: First, post-surgery IOP was not validated by comparing it with intracameral IOP readings^39^ due to its invasive nature. Second, the sequence in which we measured IOP could imply certain bias in the second and third IOP measurements: repeated tonometry may induce changes in the anterior chamber volume and thus, in the registered pressure. AlMubrad *et al*.^40^ found a statistically significant IOP reduction (1.5 ± 1.2 mmHg; *p* < 0.05) on subsequent measurements performed with a non-contact tonometer (Topcon CT80) after GAT in 65 patients. Gaton *et al*.^41^ recorded a significant IOP decrease between first and second successive measurements with GAT (15.94 mmHg vs 14.9 mmHg, *p* < 0.0001) in 70 glaucomatous eyes. We believe that repeated contact of any external force with the eye may produce occasional IOP fluctuations. This would lead to significant underestimations that could be transcendent regarding glaucoma diagnosis. However, other considerations should be taken into account, such as IOP levels beyond the normal range or CB. A third source of bias in our study could be related to IOP diurnal fluctuation in time. Baseline IOP could be unbalanced across measurements after 3 months even if measurements are taken at the exact same time^42^. Further research could determine whether CT performs accurately.

Regarding variables that could influence our measurements, CRF showed a significant correlation for all the tonometers pre- and post-surgery. As in other reported studies^33,43^, CRF decreased after both procedures but mainly in LASIK as opposed to PRK. It is evident that PRK is less invasive than LASIK, which implies that CB properties are better conserved. We believe this could be the reason why CT overestimates IOP before surgery and performs less accurately in PRK corneas. This would coincide with our clinical findings for CT1 and CT2pre, which were not useful in non-operated corneas since they overestimated IOP measurements.

The posterior corneal shift after LRS procedures has been widely evaluated to detect possible ectasia^44–46^. However, no previous studies have specifically addressed how PCC changes could influence GAT IOP readings after LRS procedures. In 50 normal subjects, Firat *et al*.^47^ found that anterior and posterior curvature values and corneal volume do not influence IOP readings made with GAT. These results coincide with our pre-surgery results in which no IOP reading of any tonometer was correlated with PCC, simK or VOL in 102 normal patients. In the post operated subgroups, PCC and VOL also did not seem to influence IOP readings. We believe this could be related to posterior corneal curvature changes recovering three months after surgery^46^, which was the time our measurements were taken. On the other hand, CCT, Max. abl, and PTA could explain the differences among GATpre, CT1 and CT2post in the LASIK group. As more corneal tissue is removed in high myopia in the anterior stroma, it is expected that CB is more altered. Therefore, a bias could be expected for all the tonometers, which would have a significant impact on GAT readings, compared to the PRK group.

Both pre- and post-surgery tissue characteristics should be taken into account when IOP measurements are considered in post LRS corneas. Although we could expect post-surgery measurements to be close to those prior to surgery, a range of known variability (as we find in GAT with normal corneas) can be expected because tonometry is not personalized. In addition, the most accurate options for measuring IOP in LRS patients are not usually available or accessible to all ophthalmologists. This new simple and affordable option could solve a problem that has not yet been solved and make it available for universal use. Notwithstanding, new studies will be necessary to confirm the data analysis, make comparisons with other tonometers, and verify whether CT could also be used in patients with hypermetropic LRS, keratoconus, or after corneal transplantation.

Nevertheless, our device has demonstrated good agreement between GAT and CT1post in the LASIK subgroup, and thus minimizes the effect of the loss of central tissue in this type of surgery. The IA/IE results also indicate that there were no significant differences between observers, and therefore it could be a reproducible and convenient alternative for any ophthalmologist, and suitable for a currently very frequent and specific patient profile^10,11^. In conclusion, we have designed a new version of the applanation tonometer that could be used after LASIK instead of the current tonometer reference. This provides a new applanation tonometry option that is appropriate for supporting the diagnosis of ocular hypertension in this subgroup of patients.

## Supplementary information

Supplementary material.

## Data Availability

The datasets generated and/or analysed during the current study are not publicly available due to exclusive rights to intellectual property of the new “CT” convex tonometer secured by a Spanish patent filed P201631280, but are available from the corresponding author upon reasonable request.
