# Supplementary material for: New applanation tonometer for myopic patients after laser refractive surgery
Source: Sci Rep. 2020 Apr 27;10:7053. doi: 10.1038/s41598-020-64013-4 (PMC7184562; doi:10.1038/s41598-020-64013-4)
Supplement: Supplementary file 1 — Supplementary material. [file 41598_2020_64013_MOESM1_ESM.pdf]

New applanation tonometer for myopic patients after laser refractive surgery

Authors: Maria Iglesias MD[1*], Francisco Yebra[2**], Bachar Kudsieh MD PhD[3], Andrea Laiseca MD[1], Cristina Santos PhD[4**], Jeroni Nadal MD PhD[1], Rafael Barraquer MD PhD[1,5], Ricardo P Casaroli-Marano MD PhD[6]

Affiliations:

1. Instituto Universitario Barraquer, Barraquer Ophthalmology Centre, Barcelona, 08012, Spain.
2. Department of Physics, University of Vigo, 36310, Galicia, Spain.
3. Department of Ophthalmology, Hospital Universitario Puerta De Hierro, 28222, Madrid, Spain.
4. Unitat Antropologia Biològica, Department Biologia Animal, Biologia Vegetal i Ecologia, Universitat Autònoma de Barcelona (UAB), Barcelona, 08193, Spain.
5. International University of Catalunya (UIC), Barcelona, 08017, Spain.
6. Department of Surgery, School of Medicine and Hospital Clinic de Barcelona, University of Barcelona (UB), Barcelona, 08036, Spain.

* Corresponding author:
Maria Iglesias
***Email: mariaiglesiasalvarez@gmail.com

Abbreviations used in the manuscript.

b	Regression coefficient
CB	Corneal biomechanics
CC	Calibration cornea
CCT	Corneal central thickness
CH	Corneal hysteresis
CI	Confidence interval
CPP	Contact pressure profile
CRF	Corneal resistance factor
CT	Convexly shaped tonometer
dpts	Diopters
FEA	Finite element analysis
GAT	Goldmann applanation tonometer
IA	Intra-observer error
ICC	Intra-class correlation coefficient
IE	Inter-observer error
IOP	Intraocular pressure
ICP	Initial contact pressure
LASIK	Laser assisted *in situ* keratomileusis
LRS	Laser refractive surgery
MCD	Maximum corneal deformation
Max.Abl	Maximum ablation depth
OC	Operated cornea
ORA	Ocular Response Analyser
PCC	Posterior corneal curvature
PDCT	Pascal tonometry
PRK	Photorefractive keratectomy
PTA	Percentage of ablated tissue
r	Radius
R	Correlation coefficient
S	Supplementary data
SER	Spherical equivalent refraction
simK	Simulated keratometry
TS	Topical steroids
VOL	Corneal volume
Y	Young’s Modulus
Z	Wilcoxon test
2D	Two-dimensional
3D	Three-dimensional

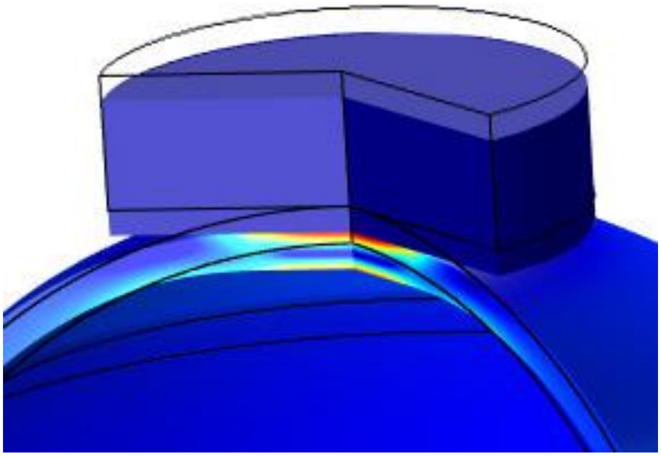

Figure S1 (supplementary data). 3D FEA simulation of a calibrated corneal antero-posterior displacement in response to a flat external surface simulating GAT.

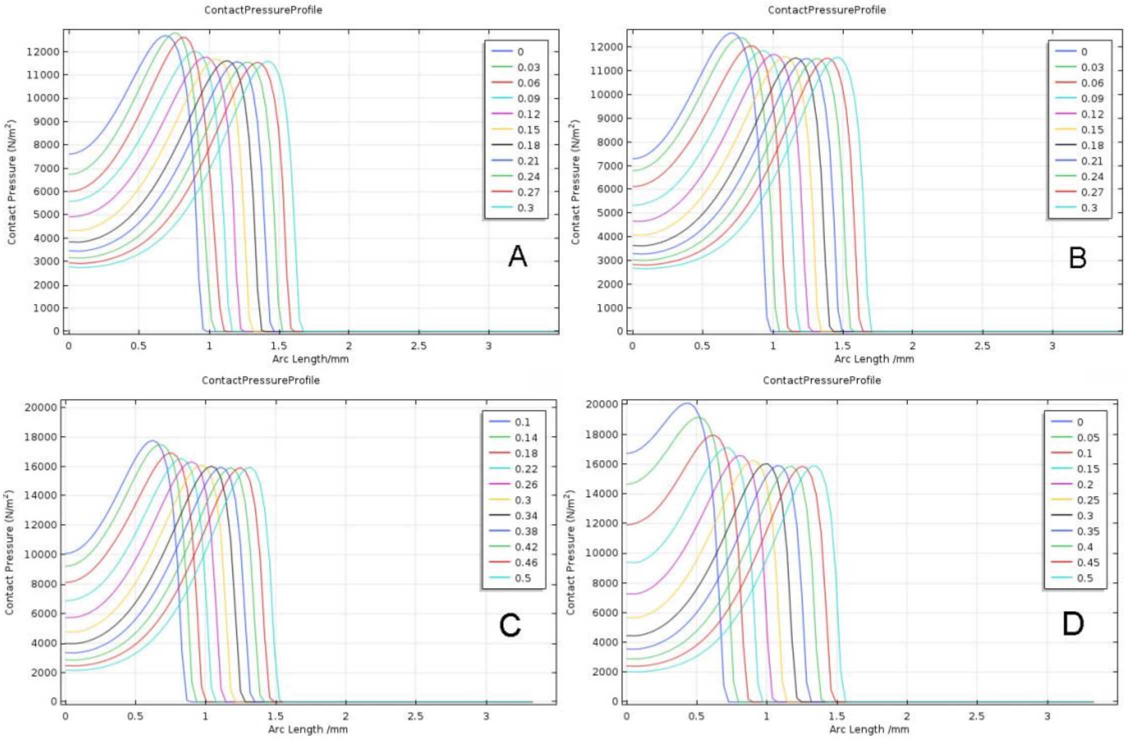

Figure S2 (supplementary data). FEA simulation of corneal antero-posterior displacement in response to GAT (A,B) and CT (C,D) taking into account different Young's Moduli (Y). Graphics correspond to force applied from the centre to the periphery of the cornea within the anterior tonometer contact surface.

Observe that there are minimal variations between the contact pressure profiles (CCT) in standard corneas with different Y. A, Y= 0.05 MPa. B, Y= 1 MPa.

However, when a CCT reduction of 50 μm is added, a large CPP variation is observed when Y is modified: C, Y =0.05 MPa. D = 0.5 MPa.

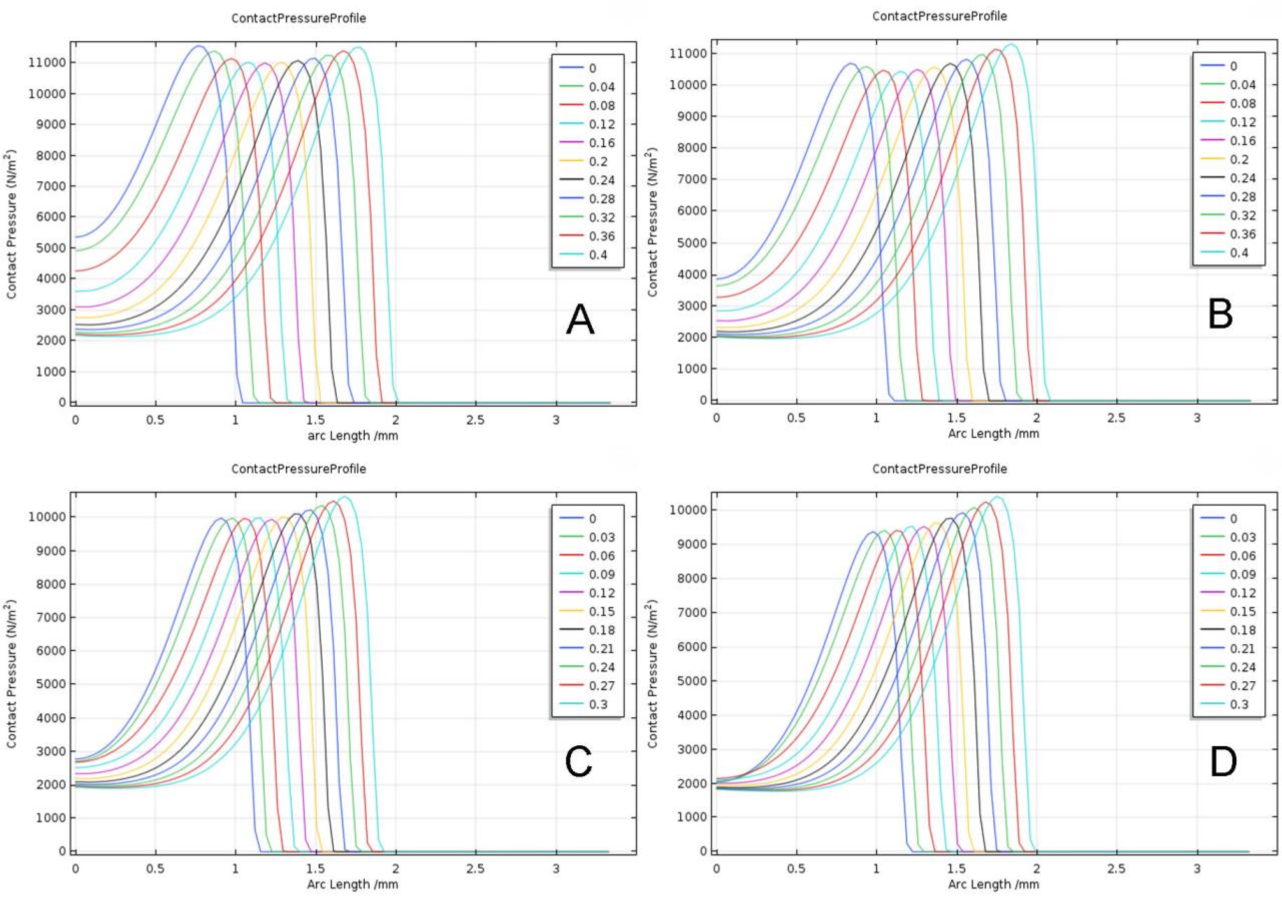

Figure S3 (supplementary data). FEA simulation of corneal antero-posterior displacement in response to GAT taking into account different reductions in central corneal thicknesses simulating different operated corneas (OC). Graphics correspond to the force applied from the centre to the periphery of the cornea within the anterior tonometer contact surface. A, OC with a 20 μm CCT reduction. B, OC with a 40 μm CCT reduction. C, OC with a 60 μm CCT reduction. D, OC with a 80 μm CCT reduction. Young's Modulus (Y) = 0.5 MPa.

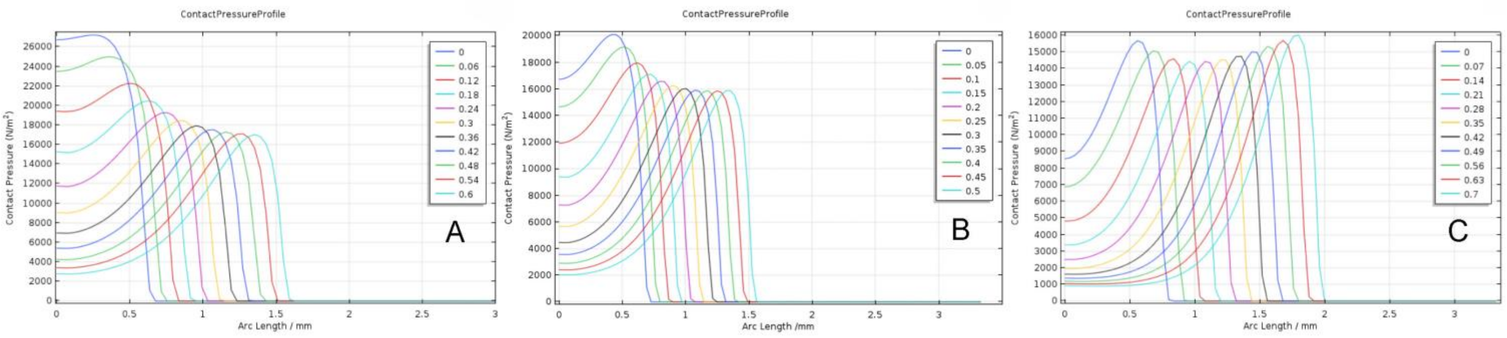

Figure S4 (supplementary data). FEA simulation of calibrated corneal antero-posterior displacement in response to CT taking into account different central corneal thicknesses (before and after LRS). Observe how the initial contact pressure (ICP) is higher before surgery (A) when compared to GAT values in Figure 1A.
A, CC. B, OC with a 50 μm CCT reduction. C, OC with a 100 μm CCT reduction. Young's Modulus (Y) = 0.5 MPa.

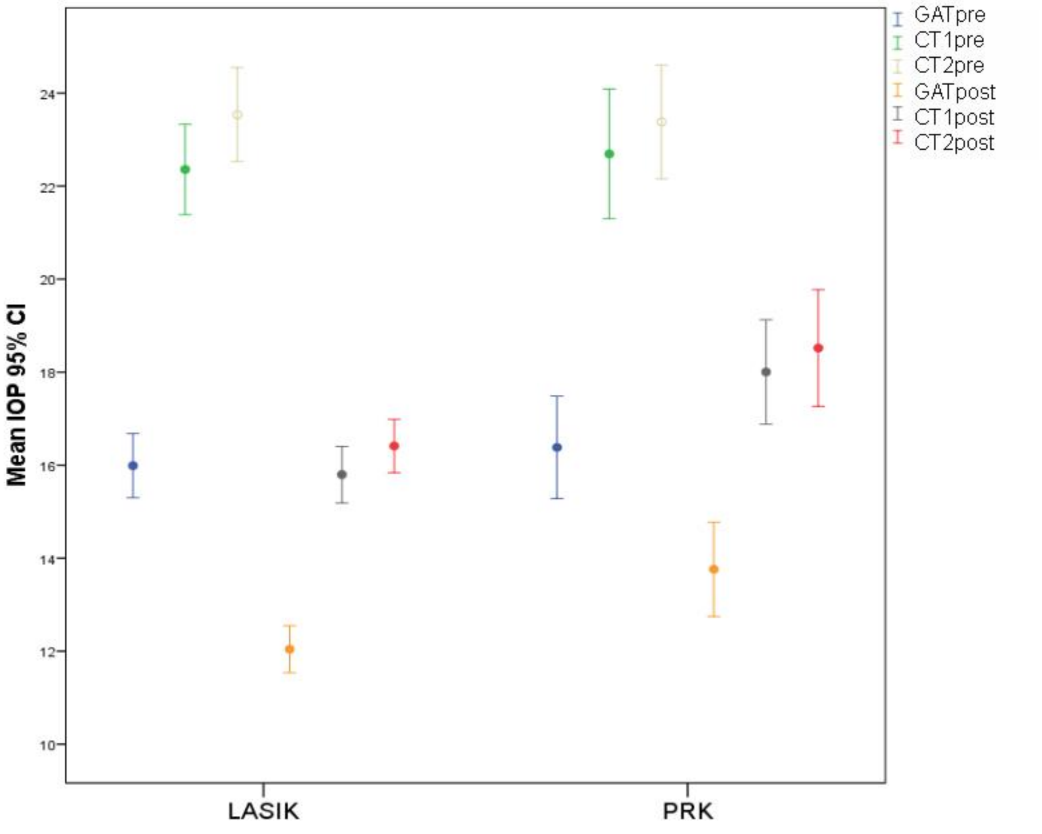

Figure S5 (supplementary data). IOP measurements with GAT, CT1 and CT2 according LASIK and PRK patients separately. GAT, Goldmann applanation tonometer; CT1-CT2, convex tonometers.
